# Covid-19 threat and coping: application of protection motivation theory to the pandemic experiences of people affected by amyotrophic lateral sclerosis

**DOI:** 10.1186/s12883-022-02662-w

**Published:** 2022-04-12

**Authors:** Shelagh K. Genuis, Westerly Luth, Tania Bubela, Wendy S. Johnston

**Affiliations:** 1 Division of Neurology, Department of Medicine, University of Alberta, 7-123 Clinical Sciences Building, Edmonton, Alberta T6G 2B7 Canada; 2 Faculty of Health Sciences, Simon Fraser University, Blusson Hall 11328, 8888 University Drive, Burnaby, BC V5A 1S6 Canada

**Keywords:** Amyotrophic lateral sclerosis, Covid 19, Pandemics, Emergency planning, Protective motivation theory, Patients, Family caregivers

## Abstract

**Background:**

People with amyotrophic lateral sclerosis (ALS) are at high risk for severe outcomes from Covid-19 infection. Researchers exploring ALS and Covid-19 have focused primarily on system response and adaptation. Using Protection Motivation Theory, we investigated how people with ALS and family caregivers appraised and responded to Covid-19 threat, the ‘costs’ associated with pandemic response, and how health professionals and systems can better support people affected by ALS who are facing public health emergencies.

**Methods:**

Data were drawn from the ‘ALS Talk Project,’ an asynchronous, moderated focus group study. Participants were recruited from regions across Canada. Seven groups met online over 14 weeks between January and July 2020. Fifty-three participants contributed to Covid-19 discussions. Data were qualitatively analyzed using directed content analysis and the constant-comparative approach.

**Results:**

Participants learned about the Covid-19 pandemic from the media. They rapidly assessed their vulnerability and responded to Covid-19 threat by following recommendations from health authorities, information monitoring, and preparing for worst-case scenarios. Adopting protective behaviors had substantial response costs, including adaptations for medical care and home support workers, threatened access to advance care, and increased caregiver burden. Participants expressed need for ALS-specific, pandemic information from trusted health professionals and/or ALS health charities. Telemedicine introduced both conveniences and costs. Prior experience with ALS provided tools for coping with Covid-19. Threat and coping appraisal was a dynamic process involving ongoing vigilance and adaptation. Findings draw attention to the lack of emergency preparedness among participants and within health systems.

**Conclusions:**

Clinicians should engage ALS patients and families in ongoing discussions about pandemic coping, strategies to mitigate response costs, care pathways in the event of Covid-19 infection, and changing information about Covid-19 variants and vaccines. Healthcare systems should incorporate flexible approaches for medical care, leveraging the benefits of telemedicine and facilitating in-person interaction as needed and where possible. Research is needed to identify strategies to mitigate response costs and to further explore the interaction between prior experience and coping. Further study is also needed to determine how communication about emergency preparedness might be effectively incorporated into clinical care for those with ALS and other medically vulnerable populations.

## Introduction

The *ALS Talk Project* (ALS Talk), a Canadian online focus group study involving people living with amyotrophic lateral sclerosis (PwALS) and family caregivers, was underway in early March 2020 when the World Health Organization declared Covid-19 to be “a global pandemic” [1]. Over the next 10 days provinces across Canada declared states of emergency with gradually tightening public health restrictions [2, 3]. At the same time, countries around the world imposed unprecedented restrictions on cross-border travel [4] and a patchwork of local ‘lockdown’ measures [5] to slow virus spread and prevent potential health system collapse. Beginning in the earliest days of the pandemic, restrictions were accompanied by messages from public health authorities. These messages encouraged individual behavior change and adoption of protective behaviors such as social distancing [6]. ALS Talk provided an opportunity to investigate the ‘real time’ perceptions and experiences of PwALS and their caregivers during the first months of the Covid-19 pandemic.

Amyotrophic lateral sclerosis (ALS) is a fatal motor neuron disease characterized by rapid, progressive motor impairment leading to severe disability and eventual respiratory failure [7]. PwALS experience an uncertain and variable disease course, with a median overall survival of 30 months after symptom onset and a 5–10% survival rate one decade after diagnosis [8, 9]. Individuals diagnosed with ALS, particularly those with compromised respiratory function, functional disability, rapid progression, or co-existing medical comorbidities, are at high risk for severe outcomes from Covid-19 infection [10–12]. Further, restricted access to in-person, multidisciplinary, medical care and public lockdowns have resulted in delayed diagnosis and treatment [10, 13–15], increased severity of ALS symptoms [16], and decreased self-perceived health status [17].

Publications exploring the impact of Covid-19 on ALS care and management focus primarily on system response and adaptation. Telemedicine’s rapid expansion in response to the pandemic has received much attention [13, 18–22], as has alternative approaches for study recruitment and data collection [13, 23–25]. A smaller body of questionnaire-based research reports increased anxiety, loneliness, and depression among people affected by ALS, both PwALS and their family caregivers [11, 26, 27]. To date, the experiences of PwALS and their families as they responded to Covid-19 and enacted protective behaviors has received limited attention.

In keeping with the recommendation to incorporate behavior change theory in investigations of infectious disease and emergency response contexts [28], we use Protection Motivation Theory (PMT) as a lens for examining how PwALS and their caregivers evaluated and responded to Covid-19 threat. This theory has been identified as an effective tool for systematic and evidence-based investigation of behavioral adaptations to Covid-19 [28], and a tool for identifying and implementing supportive strategies by health professionals [28, 29]. PMT postulates that fear-arousing communication about health threats will initiate two cognitive processes: threat and coping appraisal. People will be motivated to adopt protective behaviors recommended by health authorities when (1) they believe that the health threat is severe and that they are vulnerable to the threat; and (2) they believe that protective behaviors will effectively avert threat (response efficacy) and that they have the ability and will to adopt the recommended behaviors (self-efficacy). Further, individuals will be more likely to adopt protective behaviors if the associated response costs are low [30, 31]. PMT has been used to examine and predict protective health behaviors related to Covid-19 in general, healthy populations [32–38]. To our knowledge this theory has not been used as a lens for investigating the experiences and behavior of a medically vulnerable population responding to Covid-19 threat.

Drawing on data from ALS Talk, we investigated the response of PwALS and their caregivers to Covid-19 threat. Guided by PMT we investigated the following questions. (1) How did PwALS and family caregivers appraise and respond to Covid-19 threat in the first months of the pandemic? (2) What were the primary ‘costs’ for people affected by ALS as they adapted to life during the Covid-19 pandemic? And (3) how can ALS health professionals better support PwALS and caregivers who are facing public health emergencies?

## Methods

Data for this investigation were drawn from ALS Talk, an asynchronous, online focus group study, investigating health communication with PwALS and family caregivers throughout the disease course. The study was approved by the University of Alberta’s Research Ethics Board (Pro0008471). An amendment was approved on March 20th, 2020 allowing the addition of specific questions about participants’ experiences with Covid-19. This study focuses on data pertaining to the Covid-19 pandemic.

### Participants and recruitment

Sampling for ALS Talk was purposive. To achieve a national sample, we recruited participants in the four Canadian provinces with the largest populations (British Columbia (BC), Alberta (AB), Ontario (ON), and Quebec (QC)), as well as in two smaller provinces (New Brunswick (NB) and Nova Scotia (NS)) representing Canada’s Atlantic regions. We recruited PwALS and family caregivers via (i) clinic staff at multidisciplinary ALS clinics; (ii) mailouts/emails from the Canadian Neuromuscular Disease Registry (CNDR) [39]; and (iii) social media posts, digital newsletters, and/or emails from provincial and national non-profit ALS Societies.

Participants were required to be over 18 years of age, able to communicate in written English, and have a formal ALS diagnosis [40] or be a family member providing care for someone formally diagnosed with ALS. Family members who had cared for a PwALS in the past were permitted to participate. PwALS/caregiver dyads were not required. All qualifying volunteers were invited to participate. Caregivers included spouses, partners, siblings, and adult children of PwALS. There were separate focus groups for PwALS and family caregivers living in BC, AB, and ON. Due to low study enrollment in QC, NB, and NS (representing Canada’s eastern provinces), PwALS from these provinces participated in a single focus group. There were insufficient numbers for a caregiver focus group from these regions.

All ALS Talk participants in the seven focus groups (*n* = 100) were invited to participate in an optional discussion thread about Covid-19. People who participated in the Covid-19 discussion thread or who made comments about the pandemic at any point within the focus groups were included in the Covid-19 participant subset (*n* = 53).

### Design and data collection

We used online, asynchronous focus groups, a dynamic digital bulletin board research method [41, 42] to accommodate participants’ medical needs, provide time for reflection, facilitate the use of augmentative and alternative communication aids as needed, and allow participation from dispersed geographic locations [43–46]. Participants interacted in moderated discussions using the itracks™ platform [47]. itracks™ offers text (via typing or using eye-gaze software), video, and audio-based discussion in a threaded web forum structure. Participants used a web browser or the itracks™ app to access their focus group from locations of their choosing.

Discussions within each focus group occurred over 14 weeks, with an optional topic available in weeks 15–16 (Table 1). Topics were introduced every two weeks as new discussion sections within the platform. Topic-specific question threads were added weekly to stimulate ongoing discussion. Participants were notified by email when new questions were posted to the itracks™ platform. They read questions and posted responses to the group discussion at their convenience. For each question, participants were required to post an initial response before they could read and respond to other focus group participants. There was no interaction between different focus groups.

**Table 1:** Focus group discussion topics

Topics	Weeks	Discussion description
Intro		Register, ‘welcome to the focus group’, demographic survey introductions
1	1–2	Communication around the time of ALS Diagnosis
2	3–4	Talking about ALS changes
3	5–6	Seeking information outside the health care system
4	7–8	Research participation; complementary and alternative therapies
5	9–10	Planning for future medical care
6	11–12	Conversations about death & dying
7	13–14	Improving ALS communication and support
Optional	15–16	Participation in observational research and data sharing

Each discussion topic was actively moderated for its two-week duration by research associates with expertise in patient-oriented research and qualitative research methods (SKG, WL). Moderators stimulated further input and encouraged group interaction by responding to participant posts with probing questions. Questions and ensuing discussions remained available for participant input for the duration of the focus group. All focus group questions were optional; however, participants were encouraged to post at least weekly.

Focus groups were initiated on January 7th, 2020 (AB, ON) and March 11th, 2020 (BC, eastern provinces). The online platform was open for participant discussion until May 26th, 2020 (AB, ON) and July 14th, 2020 (BC, eastern provinces). The discussion thread pertaining to Covid-19 was posted on March 21st, 2020 (AB, ON) and on April 3rd, 2020 (BC, eastern provinces). Participants also commented on the pandemic within other discussion topics. Where appropriate, moderators asked probing questions about participants’ pandemic-related experiences. For example, within discussion topic 7, ‘Improving ALS communication and support,’ we asked participants to reflect on their experiences with telemedicine introduced by health professionals and clinics in response to the pandemic.

### Analysis

Written transcripts of focus group discussions were created automatically by itracks™. Focus group contributions provided via videorecording within itracks™ were transcribed verbatim by a professional transcriptionist and verified by a team member (SKG). We used Nvivo 12™ to facilitate data organization, identification of themes, and coding. Discussion and comments pertaining to Covid-19 were identified during analysis of the focus group data. The Covid-19 data subset was separately analysed using directed content analysis guided by theory [48] and the constant-comparative approach.

Following an initial line-by-line reading of the Covid-19 data, we developed a preliminary code book representing primary themes found in the data and the primary elements of PMT (SKG, WL). The codebook was verified by an expert ALS clinician/researcher (WSJ). During analysis, themes and definitions used in the codebook were refined in response to further distinctions found within the data. Previously coded data were re-analyzed as relationships were found between primary themes and elements of PMT and as the codebook was refined. For example, the theme, ‘impact on caregivers,’ was identified as a sub-theme of ‘response costs.’ All data were independently coded by two research associates (SKG, WL) and then discussed to consensus where coding differed.

Descriptive statistics were used to summarize participant characteristics. We conducted a matrix coding query within Nvivo 12™ to identify the discussion threads where Covid-19 data were posted.

## Results

### Demographics

Fifty-three ALS Talk participants contributed to the Covid-19 data subset (Table 2). Forty-three (81%) posted comments in the Covid-19 discussion thread. Forty-five (85%) posted reflections on the pandemic in the context of other discussion topics. For example, when discussing Topic 6, ‘Conversations about death & dying,’ a participant noted the influence of the pandemic on plans to visit a first grandchild before death: “They live in Vancouver, which is tough now with Covid for travelling” (P10, PwALS).

**Table 2:** Participant characteristics

Characteristics	ALS Talk participants *n* = 100		COVID-19 participant subset *n* = 53	
**Age**				
18–29	3	3%	0	0.0%
30–39	4	4%	2	3.8%
40–49	14	14%	7	13.2%
50–59	25	25%	7	13.2%
60–69	31	31%	20	37.7%
70+	19	19%	14	26.4%
Unassigned	4	4%	3	5.7%
**Gender**				
Female	57	57%	28	52.8%
Male	39	39%	22	41.5%
Unassigned	4	4%	3	5.7%
**Role**				
Caregiver	49	49%	22	41.5%
PwALS	51	51%	31	58.5%
**Residence**				
Alberta	32	32%	15	28.3%
British Columbia	26	26%	19	35.8%
Ontario	33	33%	17	32.1%
Eastern provinces^a^	9	9%	2	3.8%

^a^PwALS recruited from Quebec, New Brunswick, and Nova Scotia

### Threat appraisal

The majority of participants learned about Covid-19 threat from the news media, particularly from press coverage of public health announcements. Participants highlighted local, national, and international health authorities as reliable sources of information about the emerging pandemic. Participants reported that information about Covid-19 threat from ALS health care professionals was largely absent in the early months of the pandemic. Nevertheless, participants rapidly assessed available information and determined both a high level of threat from Covid-19 and substantial vulnerability for PwALS. This is exemplified by the following: “I first heard about the virus on a news program and, within a couple of days, realised how it could impact me and my family” (P21, PwALS).

In addition to determining a high level of threat for PwALS, participants rapidly began to consider a cascade of associated threats. They quickly identified Covid-19’s potentially adverse impact on disease progression and lifespan. For example, a PwALS wrote: “Since I’m on a ventilator, [Covid-19] would be fatal for me” (P68, PwALS). Further, participants identified threats to emotional wellbeing, medical care, home support, access to advance care planning, and access to end-of-life options (Table 3).

**Table 3:** Threat appraisal

Themes	Illustrative quotations
***Threats to***	
Emotional wellbeing	• “*My husband expressed the frustration of feeling isolated and not being able to fully live and enjoy the limited time he may have left*” (P52, Caregiver)
Medical care	• “*We scheduled an appointment for an assessment as my wife’s breathing is beginning to decline. Unfortunately, the appointment has been cancelled and we are not sure when it will be rescheduled*.” (P20, Caregiver)
Home support	• “*It is concerning how vulnerable he is with the various [home support workers] coming and going to care for him*.” (P59, Caregiver)
Access to advance care planning	• “*It’s entirely possible she won’t be moved to a palliative care facility due to Covid*.” (P81, Caregiver)
Access to end-of-life options	• *“I do want to speak to a Health professional about [end-of-life options], but that is hard to arrange when we are in isolation*.” (P108, PwALS)

### Coping appraisal

Participants moved rapidly from threat to coping appraisal: “Almost immediately we decided as a family to quarantine ourselves” (P43, PwALS). They universally accepted the need for protective behaviors. Many participants believed that, regardless of the challenges, they had to “adjust as best as possible” (P88, Caregiver). Discussion within the focus groups focused on response- and self-efficacy. Analysis of participants’ coping appraisal indicated four primary themes: following recommendations from health authorities, adaptations for medical care and home support workers, ongoing information monitoring and management, and preparing for worst-case scenarios.

#### Following recommendations from health authorities

All participants adapted their behaviors in response to public health recommendations. Adaptations included social distancing, new sanitary regimes in and outside of the home, and adherence to stay-at-home mandates. “We are using more wipes for disinfecting household items, surfaces…using gloves and masks” (P128, PwALS). These adaptations influenced all spheres of participants’ lives, including social interaction, coping with day-to-day tasks, and personal and medical care.

#### Adaptations for medical care and home support

Participants agreed that telemedicine was a required, adaptive response to Covid-19 threat. They highlighted the advantages, including increased convenience and effective access to health professionals (Table 4). Although participants also expressed concerns, there was overall agreement that telemedicine should become part of the suite of care offered post-pandemic.

“*I have no doubt that emerging from these troubled times, the overall toolkit of approaches will be much richer and better balanced. Yes, we’ll get back to face-to-face consultations, yet perhaps augmented with virtual consultations.*” (P4, PwALS)

A high proportion of participants were concerned about potential Covid-19 transmission from home support workers. Participants responded by adopting extensive sanitary protocols and/or foregoing professional home support.

“*We have instituted some new policies at his home (hand washing upon entry, signs up on the doors to confirm people are feeling well, changing hand towels after each shift, allowing staff to leave early/come late to eliminate overlap and reduce unnecessary contact between people)*.” (P59, Caregiver)

When making decisions to initiate, continue or discontinue the services of home support workers, participants considered the efficacy of potential protective behaviors and assessed their personal capacity to cope with adaptive behaviors. For example, “If we really weren’t managing on our own, we would have outside helpers in with proper PPE” (P116, Caregiver).

**Table 4:** Benefits and costs associated with telemedicine

Themes	Illustrative quotations
***Benefits***	
Convenience: Travel	• *“I really like not having to travel to the clinic and sit around waiting for a long time to see the doctor.”* (P33, PwALS)
Convenience: Other	• *“[Telemedicine] is much easier than getting up, showered, dressed, and ready to drive to morning appointments.”* (P1, Caregiver)
Access to health professionals	• *“All I do is phone up and I am given a date and time when the doctor will call me.”* (P51, Caregiver)
***Costs***	
Unmet physical needs	• “*My concern is that virtual appointments can’t include updated respiratory testing to see if carbon dioxide retention is an issue yet, or a neurologist’s physical or clinical assessment and tests for progression.*” (P52, Caregiver)
Functional communication barriers	• *Usually, I prepare a full page of condition status, issues, and questions. I can’t speak but my wife would be my voice. This time, given the format [telephone], I didn’t bother*.” (P49, PwALS)
Personal interactions	• “*I definitely prefer face-to-face interaction. I think I enjoy the face-to-face interaction more than I realized*.” (P70, PwALS)

#### Ongoing information monitoring and management

As part of an ongoing effort to affirm or improve the efficacy of protective behaviors, most participants closely monitored the media for announcements by public health authorities. Participants were, however, concerned about the sufficiency of protective behaviors recommended for healthy populations. They identified their acute need for practical, ALS-specific information. This is exemplified by the following: “The ALS Society and ALS Clinic should spend more time educating ALS patients and families...general information does not work to solve our daily challenges” (P8, Caregiver). Social media was rarely mentioned and, when noted, received mixed reviews. For example, one participant “found Facebook mostly useless because of the preponderance uninformed opinions” (P4, PwALS). Another suggested that ALS clinics and/or ALS health charities might support people living with ALS by providing “information on social media…where people go daily” (P131, PwALS).

A subgroup of participants reported feeling overwhelmed and stressed by unremitting media coverage of the pandemic: “I feel like my entire life has been completely consumed by the Coronavirus [Covid-19]” (P59, Caregiver). Participants facilitated coping by developing strategies to manage their media consumption. “We keep up with information via the news media – enough to stay informed and then we turn it off, so it doesn’t become overwhelming” (P1, Caregiver).

#### Preparing for worst-case scenarios

For some participants, planning for the worst of possible outcomes and considering ways to improve self-efficacy was a means of coping with pandemic threat. “Talking through different scenarios seems both practically and psychologically helpful...preparedness (a sense of control?) to face a range of possible situations” (P52, Caregiver). Participants discussed care options if family caregivers contracted Covid-19. For example, “If I got sick, what care options are left for my spouse? I have talked to his physicians, home care nurses, and hospice about the options” (P8, Caregiver). They also discussed the importance of having current advance care directives. This was exemplified by the participant who stated that she had instituted new sanitary regimes and social distancing, and “have updated my DNR [do not resuscitate] order” (P110, PwALS).

In addition to generating urgency for advance care planning, the pandemic introduced new barriers. Several participants highlighted pragmatic challenges, including access to health professionals, and required witnesses for documentation. “I haven’t talked to any health professionals about the impact of COVID-19 on my end-of-life planning…My palliative care doctor seemed quite stressed about [Covid-19]” (P10, PwALS). For others, virtual care was a barrier.

“*My husband wants to have an eyeball-to-eyeball conversation with the neurologist about what his final weeks/months will look like…He can barely speak…A Zoom meeting would only exacerbate his difficulties communicating. Don't they understand this? For God sakes, he's dying. Give the man what he wants*.” (P122, Caregiver)

Despite these barriers, consideration of worst-case scenarios was, for some participants, an important aspect of pandemic coping.

### Response cost

Participants experienced considerable ‘costs’ as they adopted protective behaviors in response to pandemic threat. Response costs were primarily associated with social distancing, changed approaches to medical care and home support, and increased caregiver burden.

#### Costs associated with social distancing

The most prominent response costs were associated with lost in-person contact with family and friends: “Coronavirus has literally stolen my time with loved ones” (P47, PWALS). Participants described generalized frustration – “I think I will go crazy if I have to stay home for 18 months” (P130, Caregiver) – as well as specific everyday losses including recreational activities and in-person support groups. For example, “I’m just plain bored. Stuck in the house with a TV and a kindle for reading. All activities that I usually participate in are cancelled” (P18, PwALS). For many participants, “the virus accentuated the [disease-related] isolation” (P7, Caregiver).

Participants were grateful for technology-mediated, distance communication with family and friends. However, these approaches introduced new costs. These were primarily related to functional communication barriers (“I wrote on my Boogie Board [reusable writing tablet [49]] and turned it so she could see my response. It was a bit awkward because of the glare from the lights” (P21, PwALS)) and physical challenges. The latter included fatigue and maintaining a “comfortable position” (P116, Caregiver) during long video calls. Notwithstanding these costs, the following quote exemplifies the perspective of most participants: “FaceTime is a godsend for us under these circumstances…It’s better than nothing” (P38, Caregiver).

#### Costs associated with changed medical care and home support

Despite participants’ acceptance of telemedicine as a threat reduction strategy and their enthusiasm for its convenience, some participants identified associated costs. Concerns focused on physical needs, functional communication barriers, and the loss of personal interaction with health professionals (Table 4).

Participants needing assistance from home support workers identified costs associated with new sanitary protocols and/or the decision to forgo professional home support. For many participants, new sanitary protocols provoked worry about efficacy. For example, “I’m quite concerned about the [professional support workers], hoping they’re following strict guidelines, but you really have no way of telling” (P10, PwALS). Those who decided to forgo professional home support identified costs associated with compromised care (“My husband was getting 2 showers a week and now he is getting no showers at all” (P53, Caregiver)) and increased caregiver burden.

#### Increased caregiver burden

Coping with Covid-19 threat increased the caregiving burden for family members. Participants emphasized changed roles and responsibilities, additional emotional stress, and decreased opportunities for self-care.

Changed roles and responsibility included the physical care of loved ones with ALS and expanded day-to-day responsibilities. A caregiving spouse wrote, “Home cooking three meals a day, caring for my husband and trying to homeschool, as well as the financial burden due to my lay-off, has been very hard to manage” (P92, Caregiver). Caregivers also reported increased responsibility for creating and managing social interaction and diversional activities: “I have, especially in these pandemic times, so little to talk about. The last role I want additionally is ‘entertainer’” (P59, Caregiver).

Our analysis suggests an undercurrent of emotional tension for many participants as they coped with both ALS and Covid-19. For many caregivers, this was expressed by concern not only for the health of a loved one with ALS, but for their own continued capacity to provide support. A caregiver wrote, “In order to take care of my kids, I need to rely on our [professional] caregivers for my Dad. But if any of them were to get sick, everything will fall apart” (P59, Caregiver).

Finally, behavior change in response to Covid-19 severely reduced caregivers’ opportunities for self-care. Participants reported the loss of specific activities (“A big source of my stress release, tennis and exercising, was unfortunately discontinued” (P122, Caregiver)) and a sense of unrelenting responsibility. For example, a caregiver stated, “I don’t ever get a break of more than an hour, even at night” (P105, Caregiver).

### Prior experience

We identified three disease-related experiences that influenced participants’ threat and coping appraisal. First, pre-existing familiarity with vulnerability: “I am facing a disease with no cure, so the pandemic doesn’t make me panic” (P108, PwALS). Second, as a consequence of progressing disability and/or social discomfort with ALS, many participants reported prior experience with isolation: “This disease has been isolating without the virus” (P7, Caregiver). And finally, although participants highlighted the absence of Covid-19 information from ALS clinics and health charities in these early months of the pandemic, participants were familiar with resolving health-related uncertainty by seeking and evaluating information from other sources. This is exemplified by the following: “I primarily use reputable media and government agencies. A couple of sites have emerged as reputable and credible aggregators of Covid-19 data. I’m relying on national health agencies, WHO, CDC and others” (P4, PwALS).

These experiences influenced participants’ coping appraisal, particularly their evaluation of self-efficacy. For example, prior experience with ALS-related vulnerability gave participants an understanding of social distancing: “Our nearest family has always been highly protective when any of them are sick, knowing his vulnerability, so they are on high alert anyway” (P7, Caregiver). And many participants had already navigated significant lifestyle adjustments: “I am okay with self-isolation. Living with ALS I only go out a few times/week anyway” (P24, PwALS). Perhaps as a result of coping with ALS-related changes, both PwALS and caregivers adopted a factual tone as they discussed coping with pandemic-related challenges. This is exemplified by the following: “We are doing what we can…I think it’s normal to have some level of anxiety under these circumstances” (P93, PwALS).

## Discussion

Unlike studies using PMT to quantify intention for or frequency of behavior change within healthy populations faced by Covid-19 threat [33, 35, 36, 38, 50, 51], we used PMT to systematically investigate and illuminate the experiences of people affected by ALS as they navigated threat and changed their behaviors in response to the Covid-19 pandemic. We now discuss how this medically vulnerable population assessed and responded to pandemic threat in the early months of Covid-19. We also discuss pandemic-related information needs, telemedicine, emergency preparedness, and study implications for medical professionals, policy, and research.

### Pandemic threat, coping, and response costs

While health authorities sought to persuade the public of pandemic threat – communicating, for instance, hospitalizations and deaths – and the need for behavior change, PwALS and their families required little persuasion. Instead, they rapidly understood the life-threatening potential of Covid-19 and began to consider ancillary threats provoked by pandemic responses. Social distancing and changed approaches to medical care, for instance, threatened access to advance care planning and end-of-life care, critical aspects of clinical care and autonomy for PwALS [52–56]. Further, protective behaviors introduced a cascade of new challenges for this medically vulnerable population. Whereas PMT is most commonly used in health settings to explore a binary proposition involving behavior change in response to fear-arousing communication [28, 29, 57], threat and coping appraisal for people affected by ALS was a dynamic process involving ongoing vigilance and adaptation.

Further, researchers exploring Covid-19 through the lens of PMT have focused primarily on threat and coping appraisal. Response cost is treated as a general construct [33, 35, 38, 58] or ignored [36, 59, 60]. Minimizing response cost, however, inhibits the theory’s practical utility [37]. Specifically, it limits understanding and potential mitigation of pandemic-related costs for different populations. For PwALS and their families, simplistic representations of response costs belie the “painfulness of the amount of work” [31] described early in the theory’s development [30, 31]. The cost of social distancing, for example, was heightened by ALS’ short disease trajectory and low 10-year survival rate [8, 9]. PwALS lost opportunities for potentially irretrievable life experiences and personal interactions. Further, protective behaviors resulted in substantial practical and emotional costs for family caregivers.

Despite the high response costs experienced by study participants, findings draw attention to the influence of past experience on pandemic response and coping. Participants had already faced a devastating diagnosis with associated practical and existential losses [61–64]. They had confronted unmet information needs [65–67] and disease-related uncertainty [64, 68]. Further, they were familiar with seeking information outside the health care system [69–72]. These life-changing experiences appeared to give participants tools for coping with the pandemic and the substantial response costs they experienced. Our findings support preliminary research indicating that people accustomed to managing ALS [11] and those who have adopted protective behaviors in the past [51] may be more resilient when faced with other unanticipated changes.

### Information need

Much attention has focused on combating Covid-19 dis-information on social media [73–76]. However, when faced with pandemic-related uncertainties, people affected by ALS explicitly preferred information from authoritative sources. Moreover, they wanted information from sources they already trusted, specifically ALS doctors and clinics, and ALS health charities. While providing accurate information early in the pandemic was challenging [77–80], the ALS Society of Canada first posted Covid-19 recommendations for PwALS and their families in a blog post on March 17th, 2020 [81]. And, since the conclusion of data collection, information about the pandemic has been posted on an ongoing basis on the websites of ALS health charities [82, 83]. Findings support the critical value of this information. Moreover, the urgent need for ALS-specific information from trusted sources will continue as people are newly diagnosed with ALS and those affected seek to make sense of vaccines, vaccine boosters, and Covid-19 variants.

### Telemedicine

The rapid acceptance of telemedicine and the implementation of novel ways for delivering ALS care in response to Covid-19 is unprecedented [13, 21]. While telemedicine has been primarily presented as a means for overcoming pandemic-related challenges [13, 18, 20], this study draws on the experiences of those affected by ALS and offers a more nuanced picture. Participants affirmed earlier studies highlighting the practical conveniences of telephone or video appointments [18], and access to support from health professionals [84]. However, participants also expressed concerns. Most prominently, PwALS and their caregivers were concerned about the efficacy of monitoring disease progression via telemedicine and relational aspects of medical care. Almost all PwALS experience motor speech disorder with disease progression [85, 86], which exacerbates feelings of loneliness and lost social connections [87]. Focus group participants highlighted these aspects of their experience as they discussed new functional communication barriers introduced by telemedicine and a loss of personal connection with health professionals.

### Emergency preparedness

People with disabilities are disproportionately affected by health emergencies, including Covid-19 [88], and natural disasters [89, 90]. Yet, research exploring emergency preparedness for people with disabilities is limited [91, 92]. We found only two publications focusing on emergency preparedness for PwALS [93, 94]. Both articles focused on earthquake preparedness. A recent survey also asked PwALS if they had a plan if their primary caregiver became infected with Covid-19 [26]. Although our findings demonstrate that some participants engaged in crisis planning as a means of coping with the pandemic threat, findings draw attention to the absence of emergency preparedness among those affected by ALS. Further, like other neurologically impaired or disabled individuals [80, 95, 96], many study participants experienced pandemic-related disruption to medical care. This suggests that there is also a lack of emergency readiness among health care professionals, in clinics, and within the health care system.

### Implications for practice, policy, and research

Our findings have implications for clinical practice, policy, and research. First, in keeping with recommendations in the disability literature [97–99], this study demonstrates a need for emergency and disaster preparedness for PwALS and their families. Routine medical visits provide an opportunity for health care professionals to identify the changing needs of patients, apply that knowledge in the context of emergencies, and discuss personalized emergency preparedness strategies. These might include determining support systems and developing evacuation plans in the event of acute threats such as flooding, fire, or earthquake [100], as well as planning for large scale disruptions that might threaten access to required supplies, for example, power sources for life-sustaining respirators [94]. In the context of Covid-19, PwALS should be provided with information and support to mitigate their substantial risk for severe outcomes from Covid-19 [95]. This must include nimble and regular communication in response to a rapidly changing landscape of Covid-19 variants and evolving information about vaccines. ALS health charities should also play a role in the timely communication of information that will support PwALS as they seek to understand and cope with Covid-19.

Further, in light of the threats and challenges provoked by recommended protective behaviors, health care professionals should discuss pandemic coping and strategies to mitigate response costs on an ongoing basis with people affected by ALS. For example, health professionals should communicate clear care pathways in the event of Covid-19 infection and encourage proactive planning, including advance directives. They should seek to support caregivers who are dealing with both pandemic-related stress and the challenges associated with disease progression [101, 102]. Finally, health professionals and clinics should leverage the benefits and mitigate the costs of telemedicine by incorporating flexible approaches to care, with face-to-face interaction as needed and where possible.

On a policy level, our findings strengthen calls for resources and support that will mitigate response costs for PwALS and other vulnerable populations [103, 104]. Particularly as the science of Covid-19 vaccines and mitigation strategies progresses, the needs of those at greatest risk from contracting Covid-19, including those with neurological disorders, should be prioritised and addressed. One identified area of concern was for home support workers. Our study supports the need for appropriate training and safety measures for home support workers who visit the homes of PwALS and other medically vulnerable populations. Safety measures may include mandatory vaccination, sufficient personal protective equipment, and adequate staffing levels to restrict the number of homes visited by any one support worker.

Although this study takes a first step towards understanding the experiences and perspectives of PwALS and their families facing a global health emergency, it also demonstrates knowledge gaps. Better understanding of the interaction between prior experience and coping appraisal, as well as response costs may facilitate targeted strategies and programs to support PwALS who are dealing with public health emergencies, including Covid-19. Researchers should explore how health care professionals and health systems might facilitate effective coping, decrease response costs, and enact strategies to support and foster self-efficacy among people affected by ALS. Further, investigation is urgently needed to determine how communication about emergency preparedness might be effectively incorporated into clinical care for PwALS and other medically vulnerable populations. The experiences, needs, and perspectives of people with disabilities have been typically under-reported and absent from emergency planning [91, 105, 106]. Empirical study is needed to better understand the needs of neurologically impaired and medically vulnerable populations during public health emergencies, and the ways in which they can be supported by health professionals and governments.

### Strengths and limitations

This study was strengthened by its large, national sample, detailed theme description, and illustrative quotations. Communication between ALS Talk participants and moderators was established when Covid-19 emerged on the world stage and these research-based relationships continued through the initial months of pandemic adjustments. Further, reflections on pandemic adaptations were stimulated directly via a focused discussion thread and moderator probes, and they emerged organically within other focus group discussions. This prolonged, triangulated approach to data collection facilitated opportunities for participants to share and reflect on pandemic experiences, thus improving the dependability of the data. Study findings were thus strengthened by research design, methodological rigor, and strong theoretical grounding [107].

There were methodological and practical limitations to this study. First, sampling may have limited findings. ALS Talk participation required internet access and the ability to interact in an online environment. Due to ALS disease characteristics – age of onset peaks at 65 years [7] – and persisting evidence of an age-related digital divide [108], our use of online focus groups may have limited sample diversity. Further, because participants contributing to the Covid-19 data subset were self-selected among ALS Talk participants, it is possible that the experiences of those who choose to discuss the pandemic differed from other ALS Talk participants. Second, although focusing on a global pandemic, the study was conducted in a single Western country with publicly funded, provincially administered health care. In this study, we do not explore the potential impact of small variations in the timing of Covid-19 restrictions and public health communication from province to province. Third, as with all qualitative research, results cannot be generalized directly to other geographical jurisdictions or medically vulnerable populations. Concerns about access to medical care, for instance, may be experienced differently by people who lose access to effective treatments for potentially terminal diseases, such as many cancers [109]. This limitation is mitigated in part by the study’s theoretical grounding [107]. Finally, PMT was chosen as a theoretical framework following data collection. We believe that this represents both a limitation and a strength. While focus group and moderator questions did not specifically probe the primary elements of PMT, participants’ descriptions of their experiences were not constrained or shaped by questions focusing specifically on PMT.

## Conclusion

This article examines the response of people living with ALS and their family caregivers to public health messaging about Covid-19 threat. Guided by PMT we found that people affected by ALS rapidly adopted protective behaviors recommended by public health authorities. Threat and coping appraisal was an ongoing, dynamic process involving vigilance and adaptation as participants sought to improve response- and self-efficacy. Findings draw attention to the substantial costs experienced by PwALS and caregivers when adopting recommended protective behaviors and the influence of prior experience on pandemic coping. Findings also highlight the need for ALS-specific, pandemic information from authoritative, trusted sources – a need that will continue as people affected with ALS seek to make sense of vaccines, Covid-19 variants, and potentially unforeseen developments. Telemedicine was highly valued for its convenience and should be established as part of the suite of care, while retaining in-person interaction as needed and where possible. This may be particularly important for advance care planning and end of life. People in this study indicated that planning for potential Covid-19 infection and advance care was an important means of coping with pandemic threat. Finally, this study demonstrates a need for emergency and disaster preparedness for PwALS and family caregivers. Action strategies and care pathways should be designed so that vulnerable individuals and the health professionals, clinics and health systems that support them, are ready for other possible emergencies or pandemics. Study findings may have implications for other neurologically impaired and/or medically vulnerable populations who are experiencing public health emergencies.

## Data Availability

The dataset generated and/or analysed during the current study are not publicly available due to parameters of our REB application/approval but are available from the corresponding author on reasonable request.

## Abbreviations

PMT: Protective Motivation Theory
PwALS: Person/people with ALS
